# AMP-activated kinase (AMPK) regulates activity of HER2 and EGFR in breast cancer

**DOI:** 10.18632/oncotarget.4474

**Published:** 2015-06-15

**Authors:** Teraneh Z. Jhaveri, Juhyung Woo, Xiaobin Shang, Ben Ho Park, Edward Gabrielson

**Affiliations:** ^1^ Departments of Pathology and Oncology and The Sydney Kimmel Cancer Center, The Johns Hopkins School of Medicine, Baltimore, MD, USA

**Keywords:** AMP-activated protein kinase (AMPK), HER2, EGFR, cancer cell metabolism, cancer therapy

## Abstract

AMP-activated Protein Kinase (AMPK) activity retards growth of many types of cancers. Investigating effects of AMPK activation on breast cancer cell signaling and survival, we found that breast cancer cell lines with amplification and over-expression of HER2 or EGFR are 2- to 5-fold more sensitive to cytotoxic effects of AICAR, a canonical pharmacological activator of AMPK, than breast cancer cell lines lacking HER2 or EGFR overexpression. Paralleling effects on cell survival, AICAR leads to dose- and time-dependent inhibition of HER2 and EGFR in HER2-amplified breast cancer cells, with activation of AMPK and suppression of HER2/EGFR activity preceding commitment to cell death. Transfection of constitutively active AMPKα also leads to decreased HER2 and EGFR phosphorylation, reduced downstream signaling associated with these receptor tyrosine kinases (RTKs), and reduced breast cancer cell growth, confirming effects of AMPK activity on HER2/EGFR. Ensuing co-immunoprecipitation experiments demonstrated an interaction of HER2 with AMPK and an *in vitro* phosphorylation assay found that HER2 and EGFR contain sequences that are potential substrates for AMPK. Our results lead us to postulate that AMPK regulates HER2 and EGFR activity in HER2-amplified breast cancer cells and thus activation of AMPK might provide therapeutic benefit in such cancers.

## INTRODUCTION

As the critical sensor of cellular energy, AMP-activated Protein Kinase (AMPK) is considered to be the master metabolic regulator of the eukaryotic cell [1]. AMPK is activated in situations that increase the cellular AMP:ATP ratio, such as exercise, ischemia, glucose deprivation, and genotoxic and oxidative stresses [2-4]. As a serine-threonine kinase, AMPK regulates a variety of cellular metabolic pathways with direct downstream targets that include glycogen synthase [5], HMG-CoA reductase [6], acetyl-CoA carboxylase 1 (ACC1) [7-9], TSC2 [10, 11], and Raptor [12]. Through these effectors, AMPK maintains energy homeostasis by turning on catabolic pathways that generate ATP while switching off anabolic pathways that consume ATP [13].

Generally, AMPK activity appears to suppress the development and growth of cancers, overcoming growth factor signaling from oncogene activation as well as exogenous growth factors [14-16]. Accordingly, AMPK activity is reduced in many cancers, including breast cancers where AMPK activation was found to be down-regulated in 90% of tumors, and in approximately 20% of non-small cell lung cancers where the AMPK-upstream activating kinase LKB1 is genetically inactivated [14, 17]. While AMPK has been proposed as a therapeutic target in breast cancer and is recognized to inhibit many of the pathways regulated by tyrosine kinase growth factor receptors [18], evidence to date has not shown a direct mechanism for this inhibition. In this article, we describe evidence that AMPK directly phosphorylates and inhibits activity of the HER2 and EGFR tyrosine protein kinases. These important cell signaling interactions between AMPK and HER2/EGFR have implications with respect to prevention and treatment of cancer.

## RESULTS

### AMPK activation preferentially inhibits growth of HER2 and/or EGFR breast cancer

**Figure 1:**
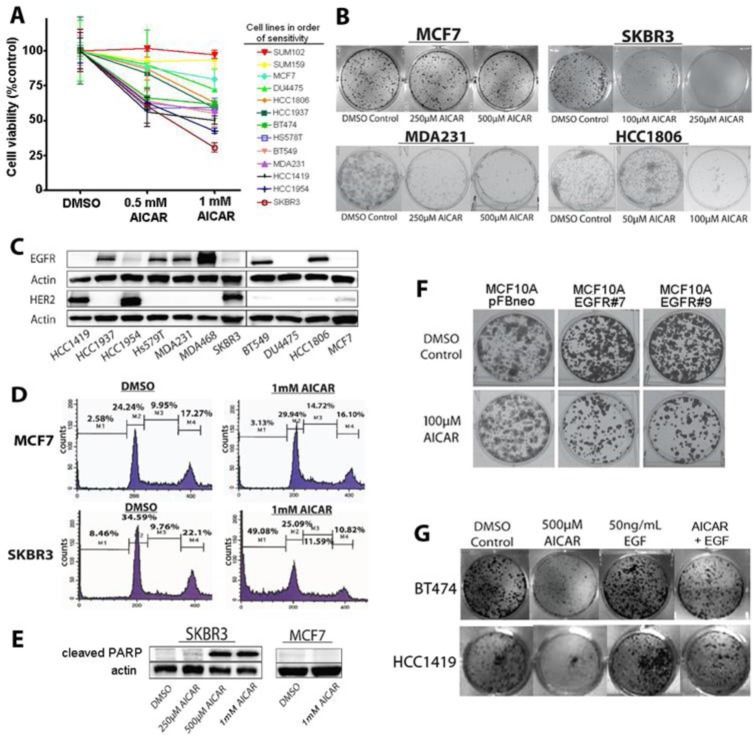
AICAR inhibits the growth and reduces survival of EGFR- and HER2-activated breast cancer cell lines **A.** MTT assay of breast cancer cell line viability after 1mM AICAR treatment for 24 hours. Data shown represent means and SEM of triplicate treatments for thirteen cell lines. **B.** Effect of AICAR treatment on colony forming efficiency of SKBR3, MCF7, MDA231 and HCC1806 cells. Survival differences parallel those of the MTT assay. All clonogenic assays were done in triplicate. **C.** Immunoblots measuring HER2 and EGFR levels in selected breast cancer cell lines. Note that cell lines sensitive to AICAR have relatively high levels of HER2, EGFR, or both. **D.** Flow cytometry measurements of cell-cycle distribution of propidium iodide-stained SKBR3 and MCF7 cells treated with either DMSO or 1mM AICAR for 24 hours. Presence of large sub-G1 populations in AICAR-treated cells is consistent with apoptosis. **E.** Western blot analysis for cleaved PARP in SKBR3 treated with indicated concentrations of AICAR (or DMSO alone) for 24 hours confirms high levels of apoptosis-mediated cell death in SKBR3 cells, but not in MCF7 cells. **F.** Colony forming efficiency of MCF10A cells treated with AICAR, comparing cells transfected with empty vector (pFBneo) to MCF10A clones with stable overexpression of EGFR (EGFR #7 and EGFR #9). Overexpression of EGFR in MCF10A cells results in increased sensitivity to AICAR. **G.** EGF treatment rescues AICAR-mediated growth inhibition of breast cancer cell lines in HCC1419 and BT474 cell lines. Representative wells from three independent clonogenic assays are shown.

To determine how AMPK activation affects proliferation and survival of breast cancer cells, we first screened a series of breast cancer cell lines for sensitivity to AICAR, the canonical activator of AMPK. As shown in Figure 1A and 1B, we observed considerable variability in the sensitivity of breast cancer cell lines to AICAR treatment, with breast cancer cell lines known to have high expression of HER2 (associated with gene amplification) or EGFR consistently showing greater sensitivity than cell lines without activation of either of these related receptor kinase subunits. Relationships between AICAR sensitivity and HER2 or EGFR activation were observed in both MTT and clonogenic assays (Figure 1A and 1B), with representative expression of HER2 and EGFR shown in Figure 1C.

We then evaluated effects of AICAR on cell cycle and cell survival by flow cytometry, comparing responses of HER2-amplified SKBR3 cells to those of HER2/EGFR-low MCF7 cells. Up to 49% of SKBR3 cells showed sub-G_1_ nuclear fragmentation after 24 hour treatment with 1mM AICAR with corresponding increases in levels of cleaved poly-ADP ribose polymerase (PARP) (Figure 1D and 1E), indicative of apoptosis in these cells. By contrast, we found minimal sub-G_1_ nuclear fragmentation and no changes in levels of cleaved PARP in similarly treated MCF7 cells.

Recognizing that complex genetic differences among these cancer-derived cell lines are not limited to the HER2 and EGFR pathways, we then sought to evaluate how sensitivity to activation of AMPK correlates with EGFR activity in a syngeneic model using MCF10A cells, a non-tumorigenic human breast epithelial cell line, and derivatives of this cell line that were engineered by retroviral transduction to stably overexpress EGFR [19]. Whereas parental MCF10A cells require high levels of EGF for proliferation, EGFR-overexpressing derivatives of MCF10A are capable of proliferating in media with low EGF [19]. As seen in Figure 1 (panel F), these two EGFR-overexpressing MCF10A clones showed reduced ability to proliferate in low-EGF media when treated with AICAR treatment. By contrast, AICAR treatment did not affect proliferation of the parental MCF10A cell line, suggesting that AMPK activation specifically targets the EGFR pathway in these cells.

We then tested whether activation of EGFR signaling by exogenous ligand can protect HER2-amplified breast cancer cells from AICAR-induced toxicity. As shown in Figure 1G, co-treatment of HER2-positive breast cancer cells with both EGF and AICAR resulted in significantly increased survival compared to AICAR treatment alone. Thus, the effects of AICAR-mediated inhibition of HER2/EGFR on cell viability can be countered by stimulating HER2/EGFR signaling through EGF ligand, consistent with the cytotoxic effects of AMPK activation being mediated mainly through inhibition of HER2/EGFR signaling.

### AMPK decreases activation of HER2 and EGFR

**Figure 2:**
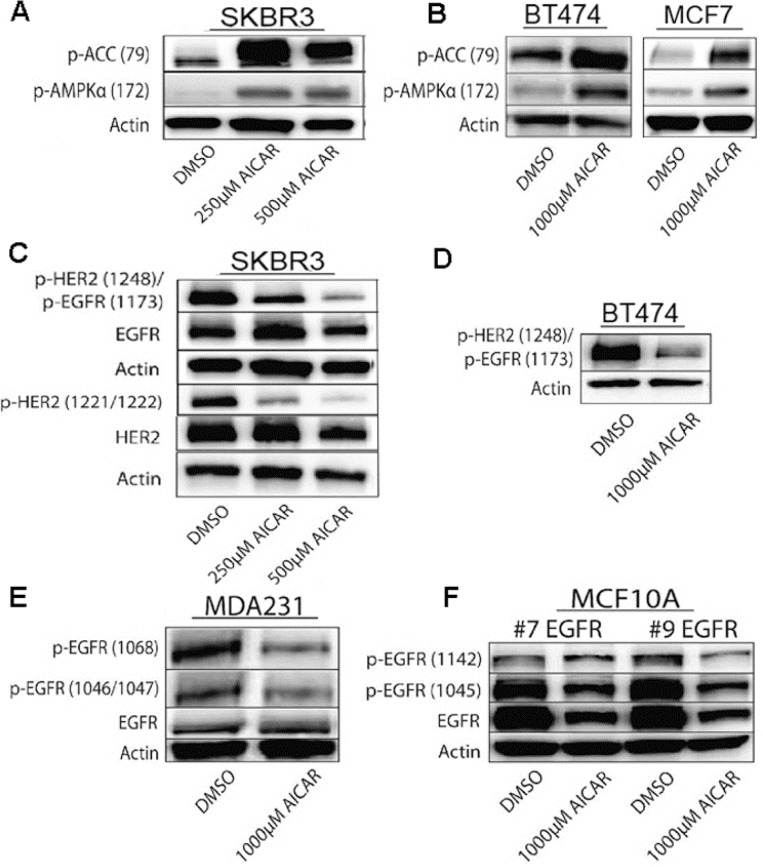
AICAR treatment activates AMPK in breast cancer cell lines **A.**, **B.** Immunoblots show AICAR-dependent activation of AMPK in SKBR3, BT474, and MCF7 cells as determined by increased phosphorylation of both AMPK and ACC, measured 24 hrs after treatments. **C.** - **F.** AICAR treatment results in decreased phosphorylation of HER2 and EGFR at auto-phosphorylation activation sites in breast cancer cells. Immunoblots show decreased levels of phosphorylated HER2 and EGFR after AICAR treatment in SKBR3, BT474, and MDA-MB-231 cells, as well as in MCF10A cells with stable overexpression of EGFR. SKBR3 cells were treated for 24 hrs and other cell lines were treated for 72 hrs.

**Figure 3:**
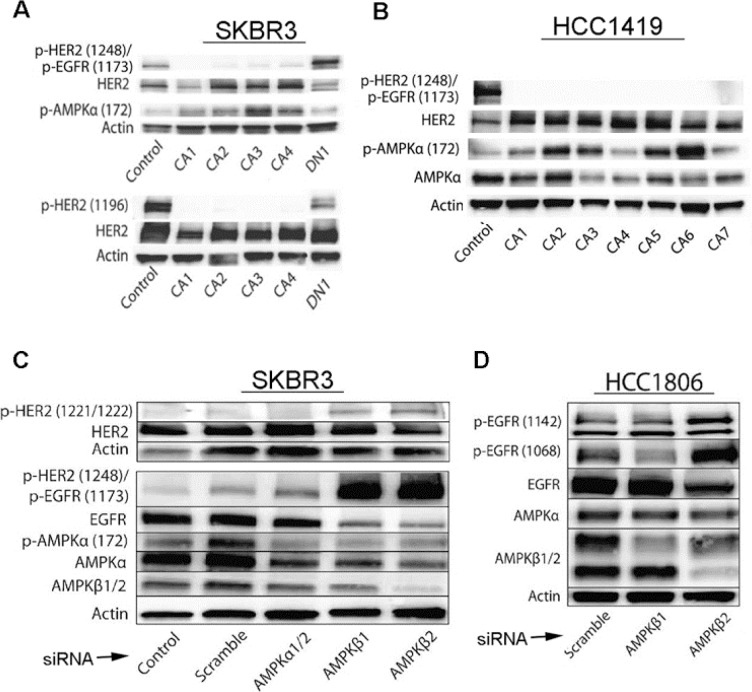
Effects of activated AMPK on phosphorylation of HER2 and EGFR is confirmed by genetic regulation of AMPK levels and activity **A.**, **B.** SKBR3 and HCC1419 cells transfected with constitutively active (CA1 – CA4) AMPKα show decreased phosphorylation of HER2 (Y1248 and Y1196) and EGFR (Y1173), whereas cells transfected with dominant negative (DN) AMPKα show increased phosphorylation of HER2 (Y1248 and Y1196) and EGFR (Y1173). pAMPK was measured to monitor efficacy of CA and DN transfections. **C.** SKBR3 cells were transfected with AMPKα1/2, AMPKβ1, AMPKβ2, or scrambled control siRNA and three days after transfection, cell lysates were evaluated by immunoblot for HER2, pHER2(Y1248)/pEGFR(Y1173), EGFR, pAMPKα(172), AMPKα, AMPKβ1/2, and pHER2(Y1221-Y1222). Note increased levels of pHER2 (Y1248)/pEGFR (Y1173) and pHER2 (Y1221-Y1222) with AMPKβ1 or AMPKβ2 knockdown, while levels of AMPKα and AMPKβ are decreased by corresponding RNAi. **D.** HCC 1806 cells were transfected with AMPKβ1, AMPKβ2, or scrambled control siRNA, and three days after transfection, cell lysates were evaluated by immunoblot for p-EGFR (Y1142) and p-EGFR (Y1068). Note increased levels of EGFR protein phosphorylation at these sites with AMPK knockdown.

Given the correlation between high HER2 or EGFR expression in breast cancer cells and sensitivity to AMPK activation, we next sought to determine how AMPK activation might affect kinase activity of EGFR and HER2. We first confirmed that AICAR's anti-growth effect was connected to reduced HER2 and EGFR signaling by correlating AMPK activation, as assessed by phosphorylation of AMPK and acetyl-CoA carboxylase, with autophosphorylation on sites of HER2 and EGFR that are linked to kinase activation [20]. As shown in Figure 2, AICAR activates AMPK at doses of less than 1 mM (Figure 3A and 3B), and this AMPK activation results in markedly diminished phosphorylation of HER2 and EGFR at activating sites (Figure 2C-2E). Extended treatment with AICAR also results in decreased levels of total HER2 and EGFR proteins in SKBR3 cells (Figure 2C).

Although AICAR is thought to be relatively specific for activation of AMPK, we sought to confirm that inhibitory effects of AICAR on HER2 and EGFR phosphorylation are mediated by AMPK by transfecting HCC1419 and SKBR3 cells with AMPKα constitutively active (CA) and dominant negative (DN) myc-tag labeled constructs [21]. Clones of cells with myc-tagged expression vectors were isolated under G418 antibiotic selection and analyzed for HER2 and EGFR phosphorylation status (Figure 3A). Similar to what we observed after AICAR treatment, AMPK-CA clones of SKBR3 and HCC1419 cells demonstrated decreased phosphorylation of HER2 and EGFR at sites associated with kinase activity (Figure 3A). Contrastingly, AMPK DN clones retained low phosphorylation levels of HER2 and EGFR. As an additional assessment of the link between AMPK activation and HER2/EGFR inhibition, we transfected cells with siRNA specific to α- and β- subunits of AMPK. (Functional AMPK is composed of α, β, and γ subunits.) We observed AMPK β siRNA to be particularly efficacious for knockdown of AMPK protein in SKBR3 and HCC1806 cells (Figure 3C and 3D), and we observed increased activating phosphorylation of EGFR and HER2 after siRNA-mediated AMPK knockdown (Figure 3). Thus, genetic modulation of AMPK levels affects the activation status of HER2 and EGFR, supporting our conclusions that increased AMPK activity inhibits HER2 and EGFR activity.

### AMPK directly phosphorylates HER2 and EGFR at specific sequences

**Figure 4:**
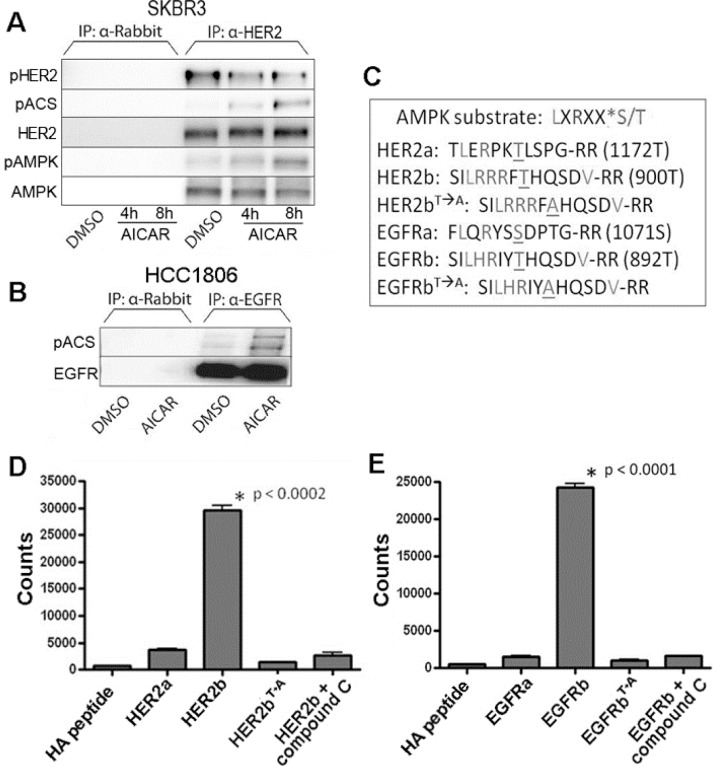
Activated AMPK phosphorylates an AMPK consensus site on HER2 and EGFR **A.**, **B.** Lysates of SKBR3 cells treated with 500μM AICAR for 4 or 8 hours and HCC1806 treated with 1 mM AICAR for 24 hours were immunoprecipitated with anti-HER2 (SKBR3) or anti-EGFR (HCC1806) and probed with an antibody specific for phosphorylation of the AMPK consensus sequence (pACS: LXRXX(p-S/T)). AMPK co-precipitates with HER2 **A.**, and levels of phosphorylated HER2 (Y1248) decrease as seen in other experiments. Conversely, phosphorylation of the AMPK consensus sequence (pACS) on HER2 and EGFR proteins increases after AICAR treatment. For both experiments, protein extracts immunoprecipitated with α-rabbit polyclonal antibody alone was used as control. **C.** Peptide sequences representing potential AMPK consensus phosphorylation sites present in HER2 and EGFR proteins. These peptides were synthesized and used in *in vitro* kinase assays. **D.** *In vitro* AMPK kinase assay using HER2 peptides (performed in triplicate) show AMPK-dependent phosphorylation of specific HER2 sequence (designated HER2b). Compound C was used as inhibitor of AMPK kinase activity, and a variant form (HER2b^T→A^) of the peptide was used to demonstrate specificity of the phosphorylation site. **E.** *In vitro* AMPK kinase assay using EGFR peptides (performed in triplicate) similarly shows AMPK-dependent phosphorylation of specific EGFR sequence (designated EGFRb). As above, compound C inhibits AMPK kinase activity, and lack of phosphorylation of a variant form (EGFRb^T→A^) of the peptide demonstrates specificity of the phosphorylation site.

In light of evidence that AMPK regulates HER2 and EGFR activity, we next questioned whether this regulation might involve direct phosphorylation of HER2 and EGFR by AMPK. Previously published data has shown an ability of the Ca^2+^/calmodulin-dependent protein kinase II to regulate activity of both HER2 and EGFR proteins by phosphorylation [22, 23], and indeed, analysis of HER2 and EGFR amino acid sequences revealed that each of these proteins also has two potential AMPK substrate consensus sequences, previously defined as LXRXX(S/T), where X represents any amino acid [12, 24]. Notably, these sites differ from those identified previously for phosphorylation by the Ca^2+^/calmodulin-dependent protein kinase II. After immunoprecipitating HER2 and EGFR in HCC1419 and HCC1806 cell extracts, we used an antibody that recognizes the phosphorylated LXRXX(S/T) AMPK consensus sequence (ACS) to show that AMPK directly binds to HER2 and EGFR and to measure phosphorylation of these AMPK substrate motifs in the HER2 and EGFR proteins (Figure 4A-4C). Phosphorylation of the LXRXX(S/T) motif increased with treatment of AICAR, indicating that AMPK directly phosphorylates HER2/EGFR proteins (Figure 4).

To confirm the ability of AMPK to phosphorylate HER2 and EGFR, and to determine which of the two possible phosphorylation sequences on each of these proteins represents the site of AMPK phosphorylation, we designed an *in vitro* kinase assay using synthesized peptides corresponding to the four potential phosphorylation sites, which we designated HER2a, HER2b, EGFRa, and EGFRb (Figure 4D). We found that sequences designated HER2b and EGFRb are readily phosphorylated by AMPK *in vitro* (Figure 4E and 4F). To confirm these sequences as the sites of AMPK phosphorylation, we synthesized peptides with modifications of the HER2b and EGFRb sequences, where the prospective phosphorylated threonines were changed to alanine: HER2b^T→A^ and EGFRb^T→A^. The inability of AMPK to phosphorylate these modified peptides (Figure 4E and 4F) confirmed that HER2 threonine 900 and EGFR threonine 892 are specific sites for phosphorylation by AMPK. Thus, our experiments collectively suggest that AMPK inhibits HER2 and EGFR activity through direct phosphorylation of specific regulatory sequences that are distinct from those sequences associated with kinase activation. Our discovery of AMPK-mediated regulation of HER2 and EGFR signaling provides a novel mechanism to explain heightened sensitivity of HER2 and EGFR overexpressing breast cancer cell lines to the AMPK activating agent, AICAR.

### Inhibition of SKBR3 xenograft growth by AICAR is associated with reduced activity of HER2

**Figure 5:**
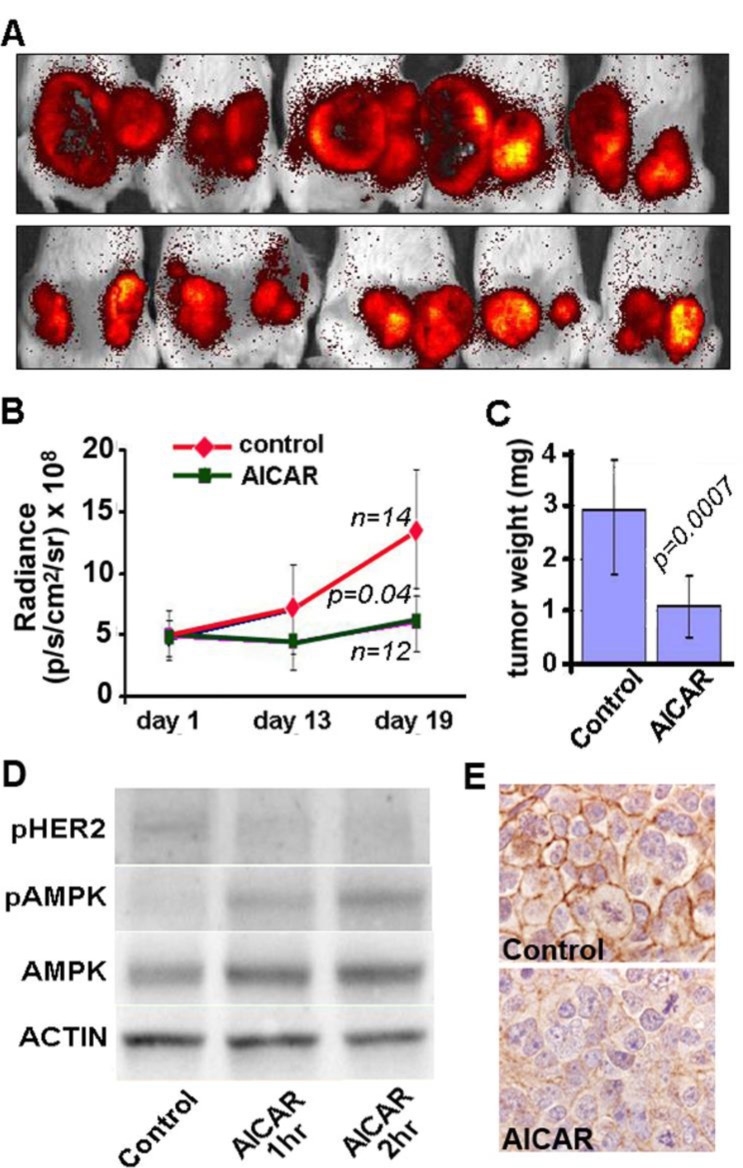
AICAR inhibits growth of SKBR3 xenografts in immunodeficient mice Growth of SKBR3 cell xenografts was monitored by measuring dT-Tomato Red fluorescence at 2, 13 and 19 days of tumor growth. Images shown in panel **A.** are of representative xenografts at 19 days for animals treated with intraperitoneal injections of either AICAR (500 mg/kg, top row) or PBS (control, bottom row). Decreased tumor growth is seen in both the measurements of fluorescence **B.** and weights of tumors explanted at the conclusion of experiments on day 20 **C.**. **D.**SKBR3 xenograft tissues show increased phosphorylation of AMPK and decreased phosphorylation of HER2 (Y1248) / EGFR (Y1173) by immunoblot of tissues explanted from animals one or two hours after treatment with AICAR (500 mg/kg). **E.** Immunohistochemistry for phosphorylated HER2 (Y1221/Y1222) shows membranous staining in tumors from untreated control animals (top) and decreased staining in tumors from animals two hours after treatment with AICAR (500 mg/kg, bottom).

To determine whether AMPK-mediated regulation of HER2 and EGFR is relevant to *in vivo* situations, we then investigated whether AICAR treatment of mice bearing SKBR3 xenografts could inhibit the growth of tumors, and whether growth inhibition would be associated with inactivation of HER2. For these experiments, NOD SCID/IL2 receptor-gamma chain knockout mice (NSG) were inoculated with SKBR3 cells that had previously been transfected with pBABE-puro-TdTomato and treated daily with a single intraperitoneal injection of AICAR. Remarkably, although AICAR has an *in vivo* half-life of only a few hours [25], this once-daily treatment resulted in significantly inhibited growth of SKBR3 xenografts as determined by measuring fluorescence in tumor-bearing mice at 2, 13 and 19 days after tumor cell inoculation and by measuring tumor weights at the conclusion of the experiments (Figure 5).

Protein extracted from tumors explanted from animals one or two hours after AICAR treatment showed increased phosphorylation of AMPK in parallel with decreased phosphorylation of HER2 at sites associated with kinase activity (Figure 5). Confirming an *in vivo* effect of AICAR treatment on HER2, we used immunohistochemistry to assess phosphorylation of HER2 at tyrosine 1221/1222, as previously reported [26]. As shown in Figure 5 (panel E) levels of pHER decreased in tumors from animals two hours after AICAR treatment, compared to control. Thus, even a sub-optimal pharmacological activation of AMPK can result in decreased HER2 activity and inhibition of growth of HER2-dependent breast cancer cells.

## DISCUSSION

In summary, we show evidence that activated AMPK negatively regulates HER2 and EGFR signaling by phosphorylating both of these proteins at novel regulatory sites, leading to inhibition of growth and survival of breast cancer cells that are dependent on these pathways. Our results thus suggest that AMPK activating agents have potential therapeutic benefit for HER2- and EGFR-dependent breast cancers.

Remarkably, HER2 and EGFR were not identified as substrates of AMPK by a chemical genetic screen that did identify twenty eight other previously unrecognized phosphorylation substrates of AMPK [27]. The inability of this screen to identify HER2 and EGFR as substrates of AMPK in this screen was likely because cell lines used in the experiments did not have high expression of these protein kinases. Both HER2 and EGFR have importance in cancer biology, however. For example, HER2 is amplified and overexpressed in approximately 15% of breast cancers, as well as some gastric, colon, endometrial, lung, cervix, esophageal and pancreatic cancers [28], and EGFR is activated by mutation in some lung cancers and also overexpressed in many lung, colon, breast, bladder, kidney, lung, and prostate cancers [29-31]. Thus, EGFR and HER2, both members of the ErBb family of receptor tyrosine kinases, are among the most significant protein kinases implicated in driving human carcinogenesis.

Key players in cancer development and growth, HER2 and EGFR have emerged as attractive targets for pharmacological intervention [30, 32]. For example, trastuzumab, a humanized monoclonal antibody to HER2 that effectively down-regulates surface HER2, is widely used in the treatment of HER2-overexpressing breast cancer [31, 33-35]. Lapatinib, a small molecule dual inhibitor of EGFR and HER2, is another therapeutic option for HER2-positive breast cancer patients [36-38], although it offers only a 4.3% response rate in patients with HER2-positive breast cancer when used as monotherapy [39-41]. While these agents have shown efficacy in the treatment of HER2 overexpressing breast cancer, not all HER2- positive cancers respond even initially to these drugs, and the development of trastuzumab and lapatinib resistance is an additional clinical problem [42]. This problem is exemplified by the number of new approved HER2 directed therapies such as pertuzumab and TDM1, which can augment trastuzumab's effectiveness and/or overcome trastuzumab resistance in HER2 positive breast cancer.

Many breast cancers, particularly those that do not express estrogen receptor, progesterone receptor, or high levels of HER2 (i.e., triple negative breast cancer (TNBC)) have high levels of expression of EGFR [43]. TNBC is often aggressive, and systemic treatment options are limited to cytotoxic chemotherapy [44]. Although anti-EGFR therapy might be expected to be efficacious for TNBC treatment, clinical trials have thus far shown no clinical benefit for a variety of anti-EGFR therapies [45]. Consequently, the development of alternative anti-EGFR therapies is of import for the treatment of TNBC.

Although much is known about EGFR and HER2 activation and subsequent signaling pathways, little is known about the regulation of EGFR and HER2 under conditions of metabolic stress. Logically, it would be expected that cells under metabolic or environmental stress would not benefit from activation of growth signaling pathways, and thus inhibition of HER2 and EGFR by AMPK is consistent with the general anti-anabolic effects of AMPK on cellular physiology. However, while AMP-activated protein kinase (AMPK) is known for its varied functions in regulating lipid, carbohydrate and protein synthesis [46], our data provide a new understanding of how AMPK also regulates cellular physiology through growth factor receptor pathways. Furthermore, our results suggest a potential therapeutic use of AMPK agonists to target HER2 or EGFR signaling in cancer.

## MATERIALS AND METHODS

### Materials

Primary antibodies specific for phospho-AMPKα (172), phospho-HER2 (1221/1222, 1248, 1196), phospho-HER2/phospho-EGFR (1248/1173), phospho-HER3 (1289), phospho-EGFR (1068, 1046/1047S, 1045), phospho-ACC (79), phospho-AMPK substrates (LXRXXpS/pT), AMPKα, AMPKβ1/2, ACC, PARP, HER2, EGFR, HER3, LKB1 and Myc-tag were purchased from Cell Signaling Technology. Anti-rabbit and anti-mouse secondary antibodies were also purchased from Cell Signaling Technology. Anti-phospho-EGFR (1142) was purchased from ECM Biosciences. Anti-Actin antibody was purchased from Sigma-Aldrich. 5-Amino-4-imidazole carboxamide riboside (AICAR), a pharmacological activator of AMPK (Toronto Research Chemicals, Toronto, ON) was dissolved in DMSO to make a 500mM stock concentration. EGF (Sigma) was prepared in a stock concentration of 100μg/ml in purified water. Compound C, an inhibitor of AMPK, was obtained from Calbiochem, and G418, used for selection of transfected cells, was obtained from Invitrogen. Control, AMPKα1/2, AMPKβ1, and AMPKβ2 siRNAs were purchased from Santa Cruz Biotechnology. All siRNA transfections were conducted in triplicate over a period of 72 hours using RNAiMax (Invitrogen) according to the manufacturer's recommendation.

### Cell culture

Human cancer cell lines HCC1937, DU4475, BT549, HCC1806, HCC1954, MDA231, HCC1419, H157, H1975, HCC827, SKMES1, U1752, Calu6 were cultured in RPMI media (Gibco) supplemented with 10% fetal bovine serum (FBS) (Gibco). BT474 was grown in DMEM/F12 media (Gibco) supplemented with 10% FBS. SUM102 and SUM159 cell lines were grown in Ham's F12 media (Gibco) with 5% FBS supplemented with 5μg/ml insulin (Gibco) and 1μg/ml hydrocortisone (Sigma). MCF7 and Hs578t cell lines were cultured in DMEM media (Gibco) with 10% FBS. SKBR3 cells were grown in McCoy's media (Gibco) with 10% FBS. MDA468 cells were grown in L15 media (Gibco) with 10% FBS in 100% atmosphere. MCF10A was grown in DMEM/F12 media supplemented with 5% horse serum (Gibco), 0.5μg/ml hydrocortisone, 10μg/ml insulin, 20ng/ml EGF, 0.1μg/ml cholera toxin (Sigma) and 100μg/ml each of penicillin and streptomycin (Gibco), and the MCF10A/EGFR cell lines were cultured as described previously (21124076). All cell lines were cultured at 37°C with 5% CO_2_.

### MTT assay

MTT assays were performed in triplicate with the CellTiter 96^®^ Non-Radioactive Cell Proliferation assay (Promega) according to manufacturer's instructions.

### Cell cycle analysis

SKBR3 and MCF7 cell lines were grown to sub-confluency in 100mm cell culture plates and treated with DMSO or 1mM AICAR for a period of 24 or 48 hours. At designated times, cells were trypsinized with 0.25% Trypsin-EDTA and fixed with 1:1 methanol:acetone. Fixed cells were stained with propidium iodide (PI, Calbiochem) for 1 hour. PI fluorescence of the samples was determined by FACSCalibur in the FL-3 channel.

### Cell viability assay

Cells were plated at a density of 2000 cells per well of a 6-well plate, and after 24 hrs incubation, treated in triplicate with variable concentrations of AICAR and EGF. Cells were cultured 8-21 days under treatment, and then fixed with 100% ethanol and stained with 0.5% crystal violet (Sigma). Cell viability was quantified by measuring absorbance with xMark™ Spectrophotometer (BioRad) at 570nm.

### Western blot analysis

Protein concentrations were determined in cell lysates (collected in TNE lysis buffer) using the Pierce BCA assay (Thermo Fisher Scientific). Equal amounts of protein from each sample were then separated by SDS-PAGE on a 10% Tris-HCl gel (Bio-Rad), transferred to a nitrocellulose membrane (Bio-Rad) and incubated overnight with the primary antibody. Most antibodies were incubated at a 1:1000 concentration, with the exception of anti-actin antibody (1:10,000). After 1 hour incubation with the anti-rabbit or anti-mouse secondary antibody, membranes were developed with SuperSignal West Femto Max Sensitivity Substrate (Thermo Fisher Scientific). For some analyses, membranes were stripped with Stripping Buffer (Thermo Fisher Scientific) and re-probed with subsequent primary antibodies.

### Constitutively active and dominant negative AMPKα

AMPKα Constitutively Active (CA) and Dominant Negative (DN) myc-tag labeled constructs in pcDNA3 (Invitrogen) plasmids [21] were generously provided by Dr. David Carling (MRC Clinical Sciences Centre, Imperial College, London, UK). *E. Coli* DH5α was made competent via CaCl2 treatment and then transformed with either control (empty pcDNA3 vector), AMPKαCA or AMPKαDN plasmid. Transformed bacteria were grown under selection on ampicillin containing LB-agar plates. DNA was isolated from bacterial colonies using the PureLink^®^ HiPure Plasmid Midiprep kit (Invitrogen). HCC1419 and SKBR3 cells were transfected in 6-well plates with plasmid DNA using Lipofectamine^®^ 2000 (Invitrogen). After 2 days, transfected cells were grown to confluency under 150μg/ml G418 selection (Invitrogen). Western blot analysis on cell lysates was used to confirm the presence of AMPKαCA /AMPKαDN using myc-tag antibody (Cell Signaling Technology).

### Co-immunoprecipitation assays

Cells were harvested in lysis buffer (50mM Tris, 150mM NaCl, 1% NP-40, 0.5% sodium deoxycholate) and supernatants incubated with rotation at 4oC with either anti-HER2 or anti-EGFR antibody overnight. Protein G-Agarose (Roche Diagnostics) was added the next day, and lysates were placed on the rotator at 4°C for 4 hours. Protein G-agarose beads were isolated by centrifugation, washed three times with lysis buffer and heated for 5 minutes at 100°C in loading buffer. Samples were run on SDS-PAGE and then probed by immunoblot for HER2, AMPKα, and an antibody specific for phosphorylation of AMPK consensus site (anti-phospho-AMPK substrates).

### *In vitro* kinase assays

AMPK (α1β1γ1) was purchased from SignalChem and assay conducted using manufacturer's protocol with Kinase Dilution Buffer VII and Kinase Assay Buffer I (SignalChem). [^32^P]- ATP was obtained from PerkinElmer. ATP and AMP were purchased from Sigma. *In vitro* AMPK inhibition was obtained with the addition of Compound C to the assay. Peptides were synthesized by the Johns Hopkins Synthesis and Sequencing Facility as follows: HA Peptide (NH_2_-YPYDVPDYA-OH), HER2#1 (NH_2_-TLERPKTLSPGRR-OH), HER2#2 (NH_2_-SILRRRFTHQSDVRR-OH), HER2#2A (NH_2_-SILRRRFAHQSDVRR-OH), EGFR#1 (NH_2_-FLQRYSSDPTGRR-OH), EGFR#2 (NH_2_-SILHRIYTHQSDVRR-OH), EGFR#2A (NH_2_-SILHRIYAHQSDVRR-OH). Samples were blotted on P81 Whatman Cellulose Paper (Sigma). Incorporation of radioisotope was measured by scintillation counting.

### Breast cancer xenografts

For xenograft experiments, SKBR3 cells at ~50% confluence were transfected with pBABE-puro-TdTomato and selected with puromycin using previously described methods [47]. NOD-SCID mice (SKCCC Animal Resources Core Facility) were then inoculated subcutaneously with 5×10^6^ cells using a Matrigel Matrix (BD Biosciences, Bedford MA). Beginning one day after inoculation, mice was randomly divided into groups for daily intraperitoneal injections of PBS (control) or AICAR (500 mg/kg). Fluorescence in tumor-bearing mice was measured at 2, 13 and 19 days after tumor cell inoculation using the Xenogen IVIS optical imaging system with an excitation filter 535nm and an emission filter 600nm, and tdTomato intensity was quantified as total photon counts using Living Image 2.50 software (Xenogen). Mice were euthanized 19 days after inoculation, and tumors were excised, weighed and prepared for protein assays or immunohistochemistry.

### Immunohistochemistry

Phosphorylated HER2 (pT1221/1222) was measured in sections of formalin-fixed, paraffin-embedded tissues explanted from xenografts described above using.

### Statistical analysis

Quantitative data were graphed and analyzed using GraphPad Prism 4 (GraphPad Software, La Jolla, CA). Error bars represent standard error unless mentioned. Student's unpaired *t* tests were used for analysis of statistical differences. Differences were considered significant at *p* < 0.05.
